# Urinary C-Peptide of Insulin as a Non-Invasive Marker of Nutritional Status: Some Practicalities

**DOI:** 10.1371/journal.pone.0022398

**Published:** 2011-07-22

**Authors:** James P. Higham, Cédric Girard-Buttoz, Antje Engelhardt, Michael Heistermann

**Affiliations:** 1 Junior Research Group Primate Sexual Selection, Reproductive Biology Unit, German Primate Centre, Göttingen, Lower Saxony, Germany; 2 Institute for Mind and Biology, University of Chicago, Chicago, Illinois, United States of America; 3 Courant Research Centre Evolution of Social Behaviour, Georg-August University, Göttingen, Lower Saxony, Germany; 4 Reproductive Biology Unit, German Primate Centre, Göttingen, Lower Saxony, Germany; Roehampton University, United Kingdom

## Abstract

Nutritional status is a critical element of many aspects of animal ecology, but has proven difficult to measure non-invasively in studies of free-ranging animals. Urinary C-peptide of insulin (UCP), a small polypeptide cleaved in an equimolar ratio from proinsulin when the body converts it to insulin, offers great promise in this regard, and recent studies of several non-human primate species have utilized it with encouraging results. Despite this, there are a number of unresolved issues related to the collection, processing, storage and transport of samples. These include: contamination of samples on collection (most commonly by dirt or faeces), short-term storage before returning to a field station, differences in processing and long-term storage methods (blotting onto filter paper, freezing, lyophilizing), and for frozen samples, transportation while keeping samples frozen. Such issues have been investigated for urine samples in particular with respect to their effects on steroid hormone metabolites, but there has been little investigation of their effects on UCP measurement. We collected samples from captive macaques, and undertook a series of experiments where we systematically manipulated samples and tested the effects on subsequent UCP measurements. We show that contamination of urine samples by faeces led to a decrease in UCP levels by >90%, but that contamination with dirt did not have substantial effects. Short-term storage (up to 12 hours) of samples on ice did not affect UCP levels significantly, but medium-term storage (up to 78 hours) did. Freezing and lyophilization for long-term storage did not affect UCP levels, but blotting onto filter paper did. A transportation simulation showed that transporting frozen samples packed in ice and insulated should be acceptable, but only if it can be completed within a period of a few days and if freeze-thaw can be avoided. We use our data to make practical recommendations for fieldworkers.

## Introduction

Nutritional status is a crucial determinant of animal survival and reproduction. Historically, the measurement of such status in free-ranging populations has been limited by the availability of non-invasive tools. In order to address such difficulties, recent studies of several non-human primate species have investigated the utility of urinary C-peptide of insulin as a non-invasive and accurate measure of nutritional status. C-peptide is produced in an equimolar ratio to insulin when the body converts proinsulin to insulin [1], and a fixed proportion of this C-peptide is then excreted in urine (urinary C-peptide; UCP), with excretion levels correlated with both plasma C-peptide levels and insulin production in humans [2]–[3]. Studies of non-human primates have demonstrated the potential of UCPs to be used as a non-invasive measure of nutritional status in at least this mammalian taxon. UCP levels have been shown to correlate with serum C-peptide levels in macaques (*Macaca spp.*) [4] and chimpanzees (*Pan troglodytes*; [5]), as well as measures of body mass and fat in macaques [4] and bonobos (*Pan paniscus*; [6]). Feeding experiments in these latter two studies have shown that UCP levels respond to dietary changes, with lower levels excreted during dieting, and higher levels during re-feeding [4], [6]. Several ecological studies have utilized UCP as a marker of nutritional status, showing that it correlates with food availability, with levels decreasing as food abundance declines (fruit, orangutans *Pongo pygmaeus* [7], chimpanzees [8]; leaves, colobus monkeys *Colobus guereza*, [9]). In addition, one published study of free-ranging rhesus macaques (*M. mulatta*) has now utilized UCP levels to monitor the direct metabolic costs of different mating strategies [10].

Despite such recent success in the utilization of this biomarker, and its promise for studies of mammalian behaviour and ecology, there are a number of potential unaddressed issues related to collection, processing, storage and transportation of urine samples for C-peptide measurement. Field workers wishing to collect biological samples and use them to measure analytes in the laboratory face many general problems. The first of these involves initial *collection and short to medium-term storage*. Urine may be shed onto the ground, in which case it may be difficult to collect samples that are completely free from dirt. In addition, animals commonly urinate and defecate at the same time, such that urine may become contaminated with faecal matter and vice versa. Such matters are important, given the potential risk that inappropriate handling of urine samples may result in biochemical changes (e.g. degradation) of compounds which may render results unreliable [e.g. 6], [11]. In some cases there are also issues of cross-contamination related to ensuring that only analytes of one origin (faecal or urinary) are measured [e.g. 12]. Following initial collection, most fieldworkers cannot return to a field station directly, as they may need to continue collecting samples and other types of data throughout the day. Even then they may only be able to return to a tent rather than a field station with electricity. This may require them to keep samples cool until processing is possible, leading to issues related to short to medium-term sample storage.

Following these initial stages, there are issues related to *sample processing and long-term storage*. Samples can be processed in several ways, including blotting onto filter paper, freezing, and lyophilization. Blotting onto filter paper can potentially be undertaken immediately on collection and is used commonly in studies of steroid hormones, so obviating the need for short-term storage of liquid samples on ice, [e.g. wild dogs *Lycaon pictus*], [ 13; Orangutans, *Pongo pygmaeus* 14]. Freezing of samples long-term for steroid hormone measurement is however the most common method employed [15], and steroid hormones typically show high stability when frozen [16]. Lyophilization is the method that manufacturers use for the long-term storage and transport of sensitive compounds such as assay antibodies and standards, but requires use of a freeze-dryer.

Finally, depending on the method utilized, there may be issues related to the *transport* of samples that are collected at field sites and then transported to laboratories for testing. In the case of particularly remote sites, this transportation process may take some time, with frozen samples taking several days to reach their destination. It may not be possible to ship samples on dry ice and/or with a courier that can maintain ice levels in the package. As such, frozen samples may experience thawing during transport. Similar freeze-thaw problems occur at field sites where power is unreliable and may occasionally fail. Collectively, the issues summarized here are well known in non-invasive behavioural endocrinology, and have been investigated mainly with respect to their effects on levels of steroid hormones [e.g. 17]–[20], [13].

The extent to which the above issues are a problem for studies of UCPs is unclear. The fact that urine contamination causing degradation may be a serious issue has been suggested by a study of captive bonobos, where a decline in levels of UCPs in samples collected from the cage floor was attributed to contamination (perhaps with the chemical cleaning products used to sterilize the enclosures [6]). Fortunately, urine-faecal cross-contamination causing co-measurement of C-peptide of different origins is not thought to be an issue, since C-peptides are not known to be excreted in faeces (though faecal-urine contamination causing degradation may still be a problem). In terms of short-term storage, some fieldworkers have kept samples on ice for periods of up to 6 hours before freezing [10], but there has been no evaluation of the effects of this, or of longer-term storage on ice, the efficacy of different processing methods, or the effects of transport. To date, the only issues evaluated have been the effects of storage on filter paper [5], [7] and freeze-thaw cycles [6] on concentrations of ape UCPs. These studies showed that human [5] and orangutan [7] samples blotted on and reconstituted from filter paper produced UCP values that were correlated with controls, while freeze-thaw cycles did not affect UCP values measured from bonobo samples [6].

Here, we address in turn the issues associated with all these aspects of urine sample collection for UCP measurement. We obtained urine samples from captive rhesus and long-tailed (*M. fascicularis*) macaques for a series of experiments in which we systematically manipulated samples and mimicked different scenarios before testing the effects of the manipulation on subsequent UCP measurements. Firstly, we tested issues related to primary field collection, by adding small particulate matter of soil and faecal material (separately) to samples and testing the effects on UCP levels. Next, we tested the effects of short- and medium-term storage on ice for up to 12 hours (short) and 3 days (medium) prior to processing in separate experiments. We then compared UCP values measured from samples placed onto filter paper and reconstituted, frozen and thawed, and lyophilized and reconstituted to assess these different processing methods. We also tested the stability of UCP levels under extended (8 months) storage on filter paper or frozen. We finished our experiments by simulating transportation on ice and assessing the effects of 24 hour freeze-thaw events. Collectively, these analyses represent a systematic attempt to address the potential practical issues a field biologist may encounter when wishing to utilize UCP in studies of ecology and behaviour.

## Materials and Methods

### Study animals and sample collection

All urine samples were collected from male and female rhesus and long-tailed macaques [4] housed at the German Primate Center. All animals were housed either as same-sex pairs or small mixed-sex groups in indoor cages and were fed twice a day with commercial monkey chow supplemented with fruits. Samples were generally collected after an animal had been observed urinating and only samples not contaminated with faeces were collected. Samples were placed on ice or in a fridge (4°C) immediately on collection and were frozen at −20°C within a few hours, except if the experiment required a different short-term storage regime (see below). All experiments described were based on samples collected non-invasively, conformed to the ABS/ASAB guidelines for the ethical treatment of animals, and were conducted in accordance with the recommendations of the Weatherall report on the use of non-human primates in research. All work was approved by the Ethical Committee of the State of Lower Saxony, Germany (permit number: 33.14-42502-04-106/09).

### C-peptide and creatinine analysis

C-Peptide concentrations were measured using a commercial C-peptide ELISA Kit from IBL International GmbH, Hamburg, Germany (Art. No. RE 53011), which we have recently validated for the measurement of C-peptide levels in macaque urine [4]. Prior to assay, urine samples were diluted between 1∶2 and 1∶10 (depending on the concentration of C-peptides) with IBL sample dilutent (Art. No. RE 53017) to bring the samples into the working range of the assay, and 100 µl of the diluted urine was then assayed using the manufacturer provided protocol. Assay sensitivity was 0.064 ng/ml. Inter-assay coefficients of variation calculated from the measurement of low, middle and high value quality controls run in each assay were 10.7%, 11.2% and 12.6%, respectively while intra-assay coefficients of variation were 6.5%, 6.7% and 5.1%, respectively.

To adjust for differences in urine concentration, C-peptide values were indexed to urinary creatinine measured according to the method by Bahr and colleagues [21]. As researchers using field samples would measure both creatinine and UCP levels from the same sample, we took the creatinine value from the same sample as the UCP value in each experimental treatment, rather than from e.g. controls. As such, our results show how indexed UCP values (those that would be of interest to field researchers) respond to the different treatments. Inter-assay coefficients of variation calculated from the measurement of low and high value quality controls run in each assay were 4.4% and 4.0% respectively, while intra-assay coefficients of variation were 2.5% and 1.9% respectively. C-peptide concentrations are presented as ng C-peptide/mg creatinine. As creatinine is also widely used when indexing other analytes such as steroid hormones [15], we also report the effects of the different treatments on creatinine levels separately, which are given as mg/ml urine.

### Collection and short- to medium- term storage

#### Contamination with soil and faeces

Since urine samples collected in the wild may be contaminated with soil or faecal matter, we tested for a potential effect of this contamination on C-peptide levels. For this, urine samples (n = 6) were portioned into three 450 µl aliquots. While one aliquot was untreated (control sample), ca. 100 mg of soil or freshly collected rhesus macaque faeces was added to the other two aliquots. After 5 min of incubation on the bench, the supernatant of each contaminated sample was pipetted off, all samples placed in the fridge (4°C) for 5 hours (to mimic storage in a cooler in the field) and subsequently stored at −20°C. Three days later, samples were thawed and analyzed as described above.

#### Short- and medium- term storage on ice

If the preferred method of processing for long-term storage is freezing or freeze-drying, then some short to medium-term storage on ice is likely to be necessary (unlike if filter-paper is the preferred method, as blotting onto filter paper can potentially be done immediately). In order to test the stability of C-peptide and creatinine levels in urine stored on ice as a function of time we undertook two experiments. In the first (short-term), we tested for a potential effect after 1, 3, 6 and 12 hours of storage on ice (n = 7 samples); in the second (medium-term), we stored samples for 24, 48 and 72 hours (n = 6 samples). For both experiments, 200 µl of each urine sample was aliquoted into Eppendorf cups. One aliquot was immediately frozen at −20°C (time 0, control sample), while the remaining aliquots were placed on ice in a thermal box for 1, 3, 6 and 12 hours (experiment 1), and 24, 48 and 72 hours (experiment 2) before being frozen at −20°C. Ice was regularly replaced over the 3 day period during experiment 2. For analysis, all samples were thawed and analyzed as described above.

### Processing and long-term storage

#### Filter paper

In order to assess the stability and recovery of C-peptide and creatinine levels from urine stored on filter paper (Grade 903, Schleicher and Schuell, Dassel, Germany), we pipetted 200 µl of freshly collected urine (n = 9 samples) on 2.5 cm squares of filter paper [14] trying to distribute the urine as evenly as possible on the paper square. Some of the remaining urine volume was then measured for C-peptide and creatinine levels as a control. The remaining urine volume of each sample was then frozen while the filter paper squares were dried on aluminium foil placed on silica desiccant at 30°C overnight. Thereafter, filter paper samples were stored individually in small plastic bags in a dark box containing a layer of silica beads to avoid contamination of samples by moisture during storage. After about 8 months these were measured and assessed against the controls measured 8 months previously. At the same time, fresh filter paper samples were prepared as above from the frozen urine, which was also re-measured at that time to act as a new control against the fresh filter paper sample. For measurement, we punched each filter paper square with a hand-held hole punch and removed 5 circles directly into an Eppendorf test tube [following: 14]. The punched circles were then eluted with 400 µl C-peptide sample diluent (see above) by placing the tubes at 4°C overnight [14], and samples were assayed the next day for both UCP and creatinine levels as described above. As the UCP diluent is yellow in colour, which interferes with the photometric creatinine measurement, we also made up the creatinine standard curve in UCP diluent to compensate for any colour effect. We confirmed that this was an appropriate correction (and did not interfere with creatinine measurement) by measuring 15 samples for creatinine both normally (i.e. dilution with distilled water; controls), and using UCP diluent to dilute samples and the standard curve (treatment). The treated samples were very strongly and highly significantly correlated with controls (Spearman's rank correlation, r = 0.994, exact p<0.001) and did not differ significantly from controls (Wilcoxon test, T^+^ = 36, exact p = 0.542). Following sample measurements, we corrected for the fact that we had only taken a portion of the filter paper square by calculating the surface area of all 5 circles, calculating what proportion of the whole 2.5 cm squares this represented, and therefore what proportion of the 200 µl original urine volume we had taken. We then corrected for this when calculating our UCP and Cr concentrations.

#### Freezing

In order to test the stability of C-peptide and creatinine levels after long-term storage of urine at sub-zero temperatures, we re-analyzed urine samples (n = 12) after 7–9 months of storage in a regular freezer (−20°C).

#### Lyophilization

Lyophilization (freeze-drying) should produce samples that are extremely stable over time, and this process is often applied for stabilizing sensitive compounds. It is also used for long-term stabilizing and storage of C-peptide standards and assay reagents (e.g. antibody, enzyme label) by the manufacturer of the C-Peptide kit. However, this method is only viable if good recovery of analyte concentrations following lyophilization can be demonstrated. We tested the recovery of C-peptides after lyophilization of urine, preparing aliquots of 250 µl in Eppendorf cups from each of 10 urine samples. All aliquots plus the remaining urine volume of each sample (control sample) were placed at −20°C until frozen. Samples (except the frozen controls) were then freeze-dried overnight. Following lyophilization, samples were reconstituted in 250 µl Millipore purified water and assayed with the matched frozen control samples as described above.

### Transport

#### Stability of frozen samples placed in an insulated container

In order to test the stability of C-peptide and creatinine levels of urine samples stored in an insulated transport container with ice packs for 3, 5, 7 and 10 days (to simulate a transport without the use of dry ice), urine samples (n = 7) were portioned into 5 aliquots of 435 µl each to which 15 µl of a 3% solution of ProClin 300 (Sigma-Aldrich Chemie, Taufkirchen, Germany), a preservation medium used by the UCP assay manufacturer to prevent microbe growth in assay standards, was added. All samples were placed in separate boxes and were immediately frozen at −20°C. After 24 hours of freezing, 4 of the 5 sample boxes were placed in the middle of an insulated container, and surrounded by frozen ice packs. The container was then left at room temperature (20–22°C). The fifth box containing control samples was left in the freezer. After three days, the first box was taken out of the container and immediately placed in the freezer. The same was done with the remaining three boxes after 5, 7 and 10 days of storage, respectively. After the experiment, all samples were assayed along with the matched frozen control samples as described above.

#### 24-hour freeze-thaw cycle

In order to evaluate the effect of thawing and storing samples at ambient temperature for one day (as to simulate power loss of a freezer, or thawing during transport) on C-peptide and creatinine levels, we pipetted 200 µl from urine samples (n = 7) into Eppendorf cups (control samples) and stored these frozen at −20°C together with the remaining urine volume. After 3 days stored frozen, samples were thawed and stored in the dark at room temperature (20–22°C) for 24 hours after which the samples were re-frozen. Samples were finally assayed along with the matched frozen control samples for creatinine and C-peptide concentrations.

### Data analysis

Data were assessed for normality using the Shapiro-Wilk test. As a number of variables were not normally distributed, and as sample sizes were small, we used non-parametric statistics. We used the Wilcoxon signed rank test to assess the effects of a single treatment, and Friedman tests for experiments containing repeated-measures over time. In these latter cases, we undertook post-hoc Wilcoxon signed rank tests to determine the time period after which effects first became significant. When these tests revealed a significant effect of the experimental treatment, we undertook Spearman's rank correlations to investigate whether treated and control values were nonetheless still correlated. All statistics were undertaken in R 2.7.0. Two-tailed tests using exact probabilities were performed, with analyses considered significant when p<0.05.

## Results

### Creatinine measurements

Generally, creatinine levels showed no or only weak effects of the different experimental treatments. Creatinine levels were statistically significantly affected by contamination with both soil and faeces, showing a decline in levels to 91±2.3% and 91±2.8% of control values, respectively (both T^+^ = 21, n = 6, p = 0.031), though such a (within assay variability) decline seems unlikely to affect results a great deal. Although creatinine levels were apparently affected by short-term storage on ice for up to 12 hours (χ^2^ = 13.9, df = 4, p = 0.008), in fact values only declined to 92±3.4% of controls, and neither medium-term storage on ice nor the 10 day transport simulation exerted any effects (all p>0.1), suggesting that the statistical significance of this result is an anomaly. Similarly, long-term freezing for 8 months, the 24 hour freeze-thaw cycle and storage at ambient temperature for 24 hours, or lyophilization, had no effects on creatinine levels (all p>0.1). The only treatment that caused more substantial declines in creatinine levels was the use of filter paper. While initial blotting onto filter paper caused only a slight and close to significant decline in values to 93±3.3% of controls (T^+^ = 38.5, n = 9, p = 0.059), long-term storage of urine on filter paper for 8 months resulted in a more substantial and significant (T^+^ = 45, n = 9, p = 0.004) decrease in levels to 74±4.0% of controls. Despite this however, no experimental treatment of any kind produced creatinine values that were not still strongly and highly significantly correlated with control values (all r>0.94, all p<0.05).

Table 1 presents a summary of the UCP results, which are considered here in more detail.

**Table 1:** Summary of UCP results.

Treatment Group	Treatment	Values by end of experiment (as % of control)	Friedman test		Wilcoxon test		Spearman's correlation (end of experiment against control)	
			χ^2^	p	T^+^	p	r	p
**Collection and short- to medium-term storage**	Soil contamination	95±8.6			16	0.313	0.829	0.058
	Faecal contamination	4±2.3			21	**0.031**	0.618	0.191
	12-hour storage on ice	87±6.4	5.2	0.269			0.964	0.003
	72-hour storage on ice	48±17.0	10.5	**0.015**			0.371	0.497
**Processing and long-term storage**	Filter paper fresh and reconstituted	50±9.7			43	**0.012**	0.946	**<0.001**
	8-month storage on filter paper	62±14.2			40	**0.039**	0.867	**0.005**
	8-month storage at −20°C	87±7.8			42.5	0.426	0.888	**<0.001**
	Lyophilization	101±3.7			39	0.922	0.988	**<0.001**
**Transport**	10-day transport simulation	37±12.6	21.26	**<0.001**			−0.143	0.783
	24-hour freeze-thaw	26±8.9			23	**0.016**	0.357	0.444

Statistically significant effects of treatment on UCP levels and significant correlations are highlighted in bold. For details see text.

### Collection and short- to medium-term storage

#### Contamination with soil and faeces

**Figure 1:**
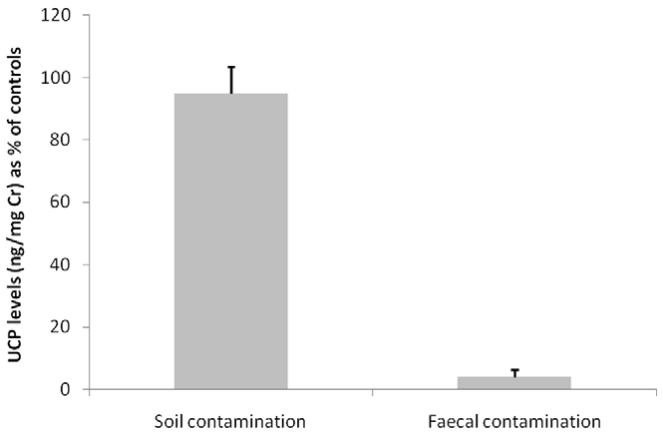
UCP levels in response to contamination with soil and faeces. Values are presented as percentage of controls.

Samples contaminated with soil declined in UCP values to 95±8.6% (mean ± SEM) of control values, which did not represent a statistically significant reduction (T^+^ = 16, n = 6, p = 0.313; Figure 1). In contrast, samples contaminated with faecal matter declined drastically to an average 4±2.3% of control values, representing a statistically significant decline (T^+^ = 21, n = 6, p = 0.031; Figure 1). UCP values were not significantly correlated with controls following faecal contamination (r = 0.618, n = 6, p = 0.191).

#### Short- and medium-term storage on ice

**Figure 2:**
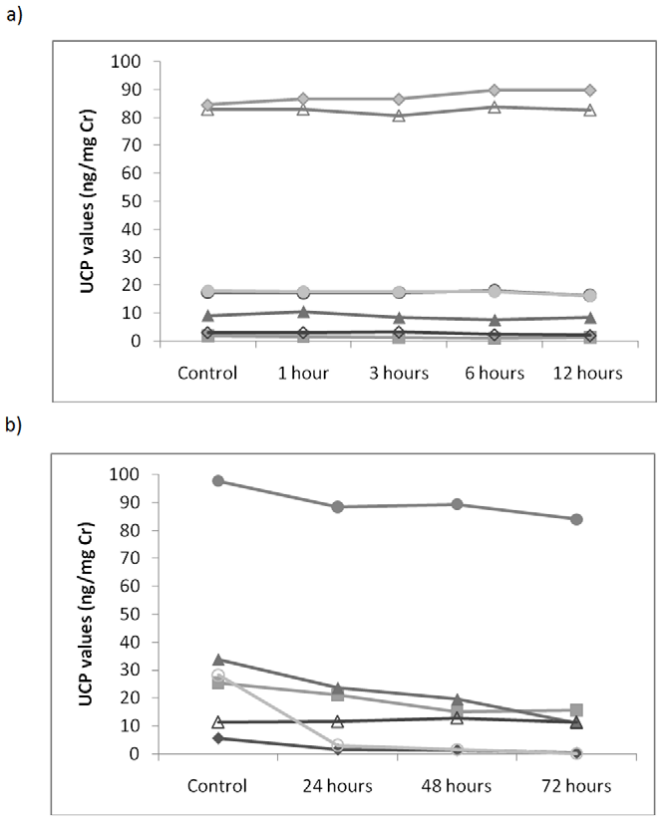
UCP levels in samples stored on ice for periods of up to: a) 12 hours; and b) 72 hours.

During short-term incubation on ice, samples did not show any decline in UCP values after 1 hour (samples 100%±3.3 of control values), but then showed a gradual decrease over the following hours (3 hours, 94±5.3%; 6 hours, 89±7.3%; 12 hours, 87±6.4% of control values). However, these changes were not statistically significant (χ^2^ = 5.2, df = 4, p = 0.269), and the relative rank order of the samples did not change over the 12 hour period (Figure 2a).

By contrast, medium-term incubation on ice resulted in a significant decline over the 3 day period tested (χ^2^ = 10.5, df = 3, p = 0.015), with samples declining to 64±14.9% of control values by 24 hours, 59±16.4% by 48 hours, and 48±17.0% by 72 hours. A post-hoc Wilcoxon test showed that the decline was already close to significant after 24 hours (T^+^ = 20, n = 6, p = 0.063). UCP values were no longer significantly correlated with controls after 24 hours (r = 0.823, n = 6, p = 0.058), with the correlation weakening substantially further by 72 hours (r = 0.371, n = 6, p = 0.497). Declines were sufficiently variable between samples (note high SEMs) that the relative rank order (from high to low) of different individual samples changed, and samples that originally had higher values than other samples now produced lower values (Figure 2b).

### Processing and long-term storage

#### Filter paper

**Figure 3:**
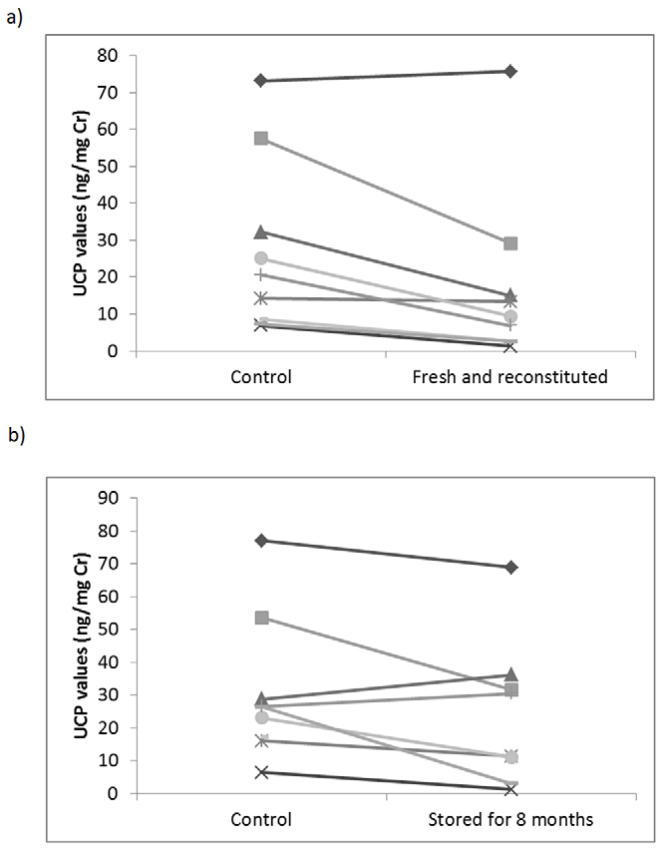
UCP levels in samples blotted onto filter paper and: a) reconstituted the next day; b) stored for 8 months.

Samples blotted onto filter paper and reconstituted the next day had significantly lower UCP values when compared to controls (50±9.7%; T^+^ = 43, n = 9, p = 0.012; Figure 3a). However, UCP values were nonetheless strongly and significantly correlated with controls (r = 0.946, n = 9, p<0.001), but did change in their relative rank order (Figure 3a). Samples blotted onto filter paper and stored for 8 months before being reconstituted showed a substantial and variable decline compared to controls (62±14.2%), which was statistically significant (T^+^ = 40, n = 9, p = 0.039; Figure 3b). However, values were still nonetheless strongly correlated with controls (r = 0.867, n = 9, p = 0.005), but again did change in their relative rank order (Figure 3b).

#### Freezing

Samples stored for 8 months frozen declined to 87±7.8% of controls, a statistically non-significant decline (T^+^ = 42.5, n = 12, p = 0.426). However, though variation in sample decline was much lower than that seen in the filter paper treatment, declines were again sufficiently variable to change the rank order of samples.

#### Lyophilization

Lyophilized samples showed no reduction in UCP values compared with controls (101±3.7%; T^+^ = 39, n = 10, p = 0.922), and due to very low variation in changes between sample concentrations as a result of this process, the rank order of the samples did not alter.

### Transport

#### Stability of frozen samples placed in an insulated container

**Figure 4:**
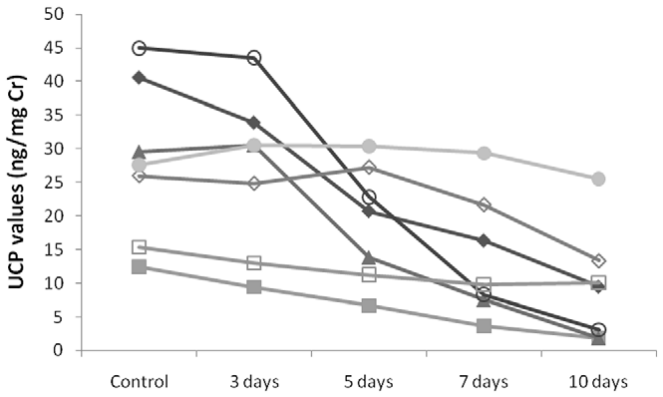
UCP levels in samples over the course of the transport simulation. Samples were surrounded by frozen ice packs in an insulated container.

Samples placed into an insulated container packed with ice packs showed increasing declines in UCP values over the experimental period when compared to controls (3 days, 93±4.6%; 5 days, 70±10.2%; 7 days, 53±12.5%; 10 days, 37±12.6% of controls; Figure 4). These declines were highly significant over the whole period (χ^2^ = 21.257, df = 4, p<0.001). Post-hoc Wilcoxon tests show that declines were not significant by 3 days (T^+^ = 22, n = 7, p = 0.219), but were close to significant after 5 days (T^+^ = 25, n = 7, p = 0.078), and significant from 7 days onwards (T^+^ = 27, n = 7, p = 0.031). Values were significantly correlated with controls after 3 days (r = 0.991, n = 7, p<0.001), but were no longer correlated with controls by 5 days (r = 0.464, n = 7, p = 0.302). Declines were sufficiently variable to cause changes in the rank order of sample concentrations by 5 days (Figure 4).

#### 24-hour freeze-thaw cycle

Samples exposed to a freeze-thaw situation declined in UCP values to 26±8.9% of control values, a significant decline (T^+^ = 23, n = 7, p = 0.016). Values were not significant correlated with controls following this freeze-thaw (r = 0.357, n = 7, p = 0.444).

## Discussion

Our study simulated a number of different realistic scenarios for fieldworkers working with urine samples for UCP measurement, and evaluated the impact of different potential issues on the concentrations of UCP measured in samples. Here, we use these results to offer practical guidance for fieldworkers for how best to treat their samples, on the understanding that in practice this will be constrained to some extent by the circumstances under which fieldworkers collect their data.

In contrast to a previous study [11], our results do not suggest that the practical issues associated with field collection, storage, processing and transport of urine have major effects on urinary creatinine levels. Only long-term storage of urine on filter paper reduced creatinine levels to lower than 90% of controls, and in all treatments, creatinine levels were strongly positively correlated with control values, indicating the general robustness of this important compound used for correcting levels of urinary analytes for urine dilution. In contrast, a number of treatments appeared to affect UCP levels indexed for creatinine. Contamination with faeces is likely to produce an enormous reduction in UCP levels, sufficient to render samples useless as they are no longer significantly correlated with controls. Whether this is due to chemical degradation processes or absorption of C-peptides into the faecal matrix is unknown. It is clear from our results, however, that contamination of urine with faecal matter must be avoided at all costs, and that no samples should be collected under such circumstances, given that some samples will inevitably not have such contamination, and will therefore appear to contain far higher levels by comparison. By contrast, contamination with dirt did not significantly reduce UCP levels. Nevertheless we recommend that samples are checked for cleanliness. If there is any particulate matter in the sample, this should be allowed to settle to the bottom for a few minutes after collection and the clean supernatant urine should be pipetted off into a new tube to minimize any potential contamination effects of dirt. This process can be repeated until the sample is clean (i.e. no new particulate matter settles out of the sample) [10]. Short-term storage on ice for up to 12 hours did not reduce UCP levels to a statistically significant degree, indicating that carrying samples in a cool environment before returning to camp for longer-term storage or processing is an appropriate way of keeping C-peptide levels stable. However, storage for longer periods on ice before processing (e.g. up to 3 days as simulated in the present study) reduces UCP levels substantially, and with sufficient variability to ensure that values are no longer correlated with controls. As such, though samples can be stored on ice for short periods of up to 12 hours in coolers, they must then be further processed without greater delay.

Of the potential further processing methods for long-term storage, lyophilization looks by far the best option if available. Recovery of values from lyophilized samples was extremely high and very consistent, with the treatment not resulting in any change to the relative rank order of samples. Once lyophilized, C-peptides should be extremely stable over long storage periods. Indeed this is the process that the manufacturer uses to store and transport the C-peptide standards and controls for the assay kit. Freezing samples also seems a reliable long-term storage method, and is likely to be more achievable for most researchers. However, although 8 months of freezing did not significantly alter C-peptide levels in our study, a few individual samples showed a decline in C-peptide levels by more than 20%, which partly resulted in a change of relative rank order of those samples that were relatively similar in original UCP levels. This method also comes with various transportation problems, which we consider below. The other method of processing for long-term storage we tested was blotting urine onto filter paper. The immediate blotting and reconstitution of samples onto filter paper significantly reduced UCP levels, but these samples, as well as those stored for 8 months, were nonetheless still significantly positively correlated with controls, consistent with results assessing values in urine samples stored on filter paper for humans [5] and orangutans [7]. Blotting onto filter paper showed the largest variation in decline in values between different samples of any processing method, with declines sufficient for individuals to change positions in their relative rank order from high to low. One potential problem involved in the process of reconstituting samples from filter paper is that discs were prone to sticking together during the process, which potentially hampers reconstitution. This effect may vary for different chemical compounds (depending on their size or solubility), as indicated by the significantly higher recovery of creatinine levels compared to UCPs. One potential solution to this problem might be to increase the amount of diluent used for reconstitution. This may be feasible for larger bodied species (e.g. apes) but may not be feasible for species such as macaques that have UCP concentrations that are often below assay sensitivity [10].

Taken together, these results show that for both long-term freezing and blotting onto filter paper, taking multiple samples from each individual and averaging is important, i.e. inter-individual differences in sample values should not be assumed to be accurate on the basis of one individual sample. It is also important to consider the type of question that a study aims to investigate – those that are reliant on measuring differences between specific individuals are more susceptible to these problems than those only interested in gross-scale (e.g. seasonal) patterns. Given that filter paper methods induced the highest degree of variation in UCP value declines, we advise the use of freezing over filter paper methods if this is possible.

For samples that are either lyophilized or blotted onto filter paper, and that can be transported at room temperature, transportation should be relatively straight-forward. For those that are frozen, there are storage and transportation issues. The assessment of the freeze-thaw cycle revealed major declines in values as a consequence of spending 24 hours at room temperature. Results from bonobos showed that a single freeze-thaw did not have such effects [6]; this suggests that it is likely to be the 24 hours at room temperature that caused the change in values. Declines were highly variable between samples, such that values were no longer correlated with controls. These results suggest that researchers must take care to ensure that freezers at field stations do not lose power – packing such freezers with ice packs and leaving only a small amount of room in which samples are stored will ensure that freezers are able to retain subzero temperatures for much longer periods in the event of a power failure. Our transportation simulation also revealed major declines in values for samples in a cooler with ice packs over periods of up to 10 days. If fast transportation of samples can be assured such that all samples still arrive frozen [as in, e.g., 10], then samples can be shipped frozen on ice packs with no issues. However, if samples are likely to be delayed during transportation (e.g. by customs authorities), or if the transportation process is likely to take more than 2–3 days, then samples should not be shipped on ice or dry ice unless with a courier that can undertake to replacing this coolant, and that can guarantee that samples will not thaw.

In sum, for fieldworkers working with urine samples for UCP analysis, we recommend: collection of samples free from contamination with faeces, that are cleaned as soon as possible for any contamination with soil; short-term storage of samples on ice (e.g. in a cooler); lyophilization within 12 hours if possible, freezing within 12 hours if not, and use of filter paper if neither method is available; and shipment of any frozen samples for periods of longer than a few days only with an assurance that they will remain frozen. Though we have tested several important issues related to the use of UCPs in field studies, it should be noted that sample sizes per experiment are not large. However, patterns were usually consistent across samples within a treatment type. The extent to which these results hold for other species is unknown, and we encourage further testing of such issues, especially in other species. If samples are treated appropriately, then urinary C-peptide of insulin measurement has great potential as a validated, sensitive and reliable method to assess the nutritional status of animals in free-ranging populations [4]–[10].
